# Reactivity of Myoglobin Reconstituted with Cobalt Corrole toward Hydrogen Peroxide

**DOI:** 10.3390/ijms23094829

**Published:** 2022-04-27

**Authors:** Koji Oohora, Hirotaka Tomoda, Takashi Hayashi

**Affiliations:** Department of Applied Chemistry, Graduate School of Engineering, Osaka University, Suita 565-0871, Japan; h_tomoda@chem.eng.osaka-u.ac.jp

**Keywords:** porphyrinoid, metalloprotein, oxidation

## Abstract

The protein matrix of natural metalloenzymes regulates the reactivity of metal complexes to establish unique catalysts. We describe the incorporation of a cobalt complex of corrole (CoCor), a trianionic porphyrinoid metal ligand, into an apo-form of myoglobin to provide a reconstituted protein (rMb(CoCor)). This protein was characterized by UV-vis, EPR, and mass spectroscopic measurements. The reaction of rMb(CoCor) with hydrogen peroxide promotes an irreversible oxidation of the CoCor cofactor, whereas the same reaction in the presence of a phenol derivative yields the cation radical form of CoCor. Detailed kinetic investigations indicate the formation of a transient hydroperoxo complex of rMb(CoCor) which promotes the oxidation of the phenol derivatives. This mechanism is significantly different for native heme-dependent peroxidases, which generate a metal-oxo species as an active intermediate in a reaction with hydrogen peroxide. The present findings of unique reactivity will contribute to further design of artificial metalloenzymes.

## 1. Introduction

Metal porphyrinoid complexes demonstrate various reactivities and physicochemical properties. Heme, an iron porphyrin complex, is known as an important cofactor in biological systems [1,2,3,4,5]. This cofactor is bound within a protein matrix to provide hemoproteins with a range of activities including catalysis, electron transfer, and binding of small ligands for various functions. A group of unique catalytic oxidation reactions are promoted by families of hemoproteins know as cytochrome P450s, cytochrome *c* oxidases and peroxidases. Inspired by these reactivities, metal complexes of synthetic porphyrinoids and their derivatives have been investigated as catalysts for oxidation reactions [6,7]. Synthetic metal complexes of porphyrinoids have been bound into a protein matrix to develop artificial activities [8]. In particular, the apo-form of myoglobin (Mb), an oxygen storage hemoprotein, is utilized for this purpose due to its simple structure.

**Figure 1:**
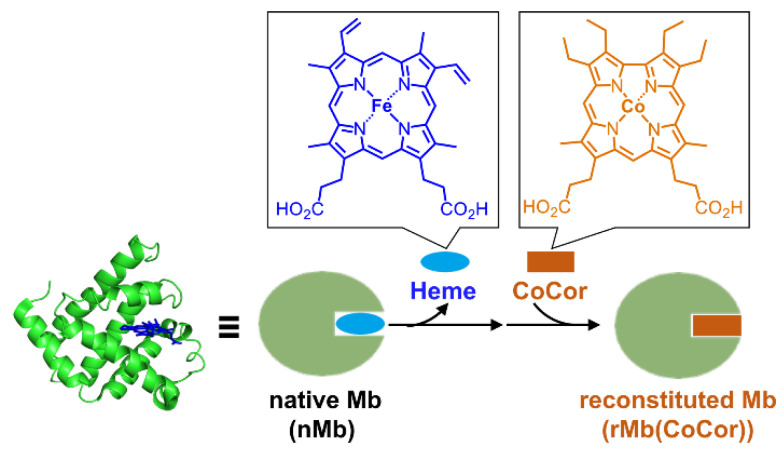
Schematic representation of reconstitution of myoglobin with a cobalt corrole complex.

Our group has recently demonstrated that Mbs reconstituted with several synthetic cofactors have much higher oxidation activity compared with native Mb (nMb) [8,9]. Fe corrole is a useful artificial cofactor to produce a reconstituted myoglobin capable of catalyzing hydrogen peroxide-dependent guaiacol (2-methoxyphenol) oxidation [10]. This catalytic activity may be due to efficient formation of the high valent species as an active intermediate because the trianionic character of corrole as a metal ligand stabilizes the high valent species, whereas porphyrin is a dianionic ligand [11]. In this investigation, Co corrole is employed as an artificial cofactor of Mb. Co corrole has been examined by several researchers as a catalyst for oxygen reduction [12], water oxidation [13,14], and hydrogen evolution [15]. However, investigations of the reactivity of Co corrole with hydrogen peroxide have been rare. Here, we report the preparation and characterization of Mb reconstituted with Co corrole (Figure 1) and investigate its unique reactivity toward hydrogen peroxide.

## 2. Results and Discussion

### 2.1. Synthesis and Characterization of a Co Corrole Complex as an Artificial Cofactor of Mb

**Scheme 1:**
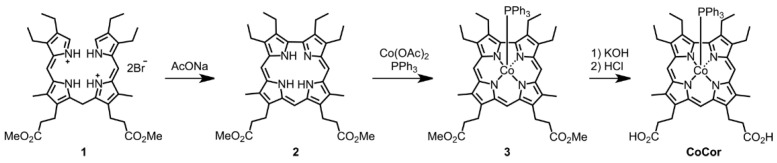
Synthesis of CoCor.

**Figure 2:**
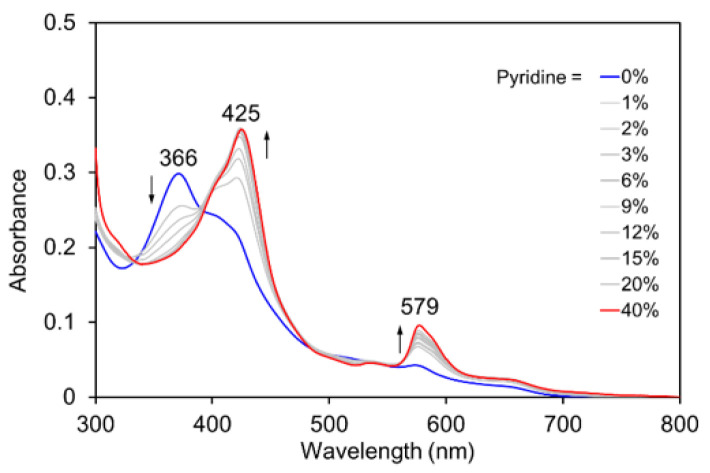
UV-vis spectral changes of CoCor upon addition of pyridine (blue line: 0% *v/v*, red line: 40% *v/v*). [CoCor] = 5 μM in MeOH containing 1–40% *v/v* pyridine at 25 °C.

**Figure 3:**
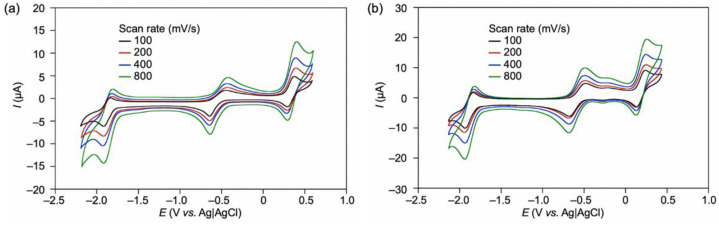
CVs of compound **3** (0.5 mM) (**a**) in DMF and (**b**) in DMF containing 5% pyridine with 100 mM TBAPF_6_ under an N_2_ atmosphere by various scan rates. Glassy carbon working electrode and Pt counter electrode were used.

The Co corrole (CoCor) used for the reconstitution of Mb was designed and synthesized according to Scheme 1. The corrole with two methyl esters of propionate side chains was prepared and insertion of cobalt was carried out using cobalt acetate in the presence of triphenylphosphine [16]. After hydrolysis of the methyl ester moieties, the target Co corrole with two propionate side chains was obtained. The ESI mass spectrum of this compound indicates the ligation with triphenylphosphine and observation of a silent ESR spectrum indicates the Co^III^ species. UV-vis spectral changes occur upon addition of pyridine to CoCor in a solution of methanol (Figure 2). The decrease in absorption at 366 nm with a concomitant increase in the absorption peak at 425 nm is similar to the previously reported coordination behavior of a Co corrole derivative [17]. This result clearly demonstrates that triphenylphosphine is replaced with another axial ligand. Since the solubility of CoCor into organic solvents is low, the redox behavior of Co corrole dimethyl ester **3** was evaluated by cyclic voltammetry (CV). Figure 3 shows the CV traces, and three redox couples are observed at –1.88, –0.55, and 0.33 V vs. Ag/AgCl, which were attributed to Co^I^Cor/Co^II^Cor, Co^II^Cor/Co^III^Cor and Co^III^Cor/Co^III^Cor^+^∙, respectively, according to a previous report [18]. Upon addition of pyridine, the changes of Co^I^Cor/Co^II^Cor and Co^II^Cor/Co^III^Cor couples are negligible, whereas Co^III^Cor/Co^III^Cor^+^∙ is shifted negatively by 0.14 V. These observations are similar to the observations made in a previous investigation of a Co corrole derivative [16].

### 2.2. Preparation and Characterization of Myoglobin Reconstituted with Co Corrole

**Figure 4:**
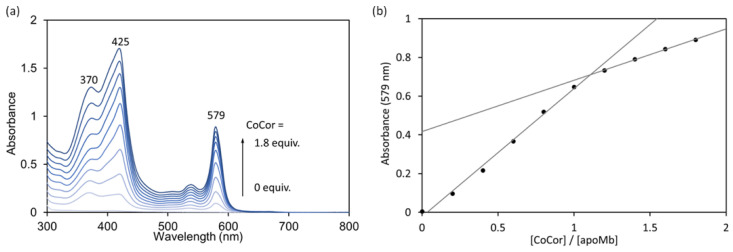
(**a**) UV-vis spectral changes upon addition of a pyridine solution of CoCor (500 μM) to a solution of apoMb. (**b**) Absorption changes at 579 nm. Conditions: [apoMb] = 20 μM in 100 mM potassium phosphate buffer (pH 7.0), [CoCor] = 500 μM in pyridine, 25 °C.

**Figure 5:**
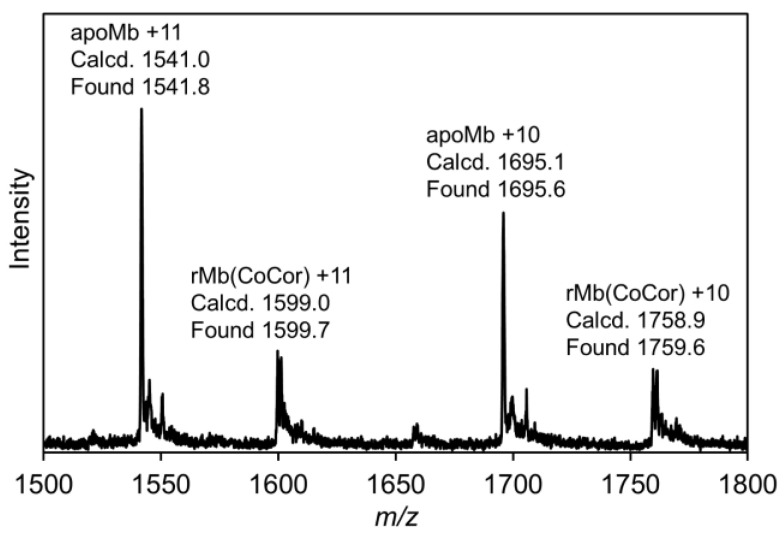
ESI-TOF mass spectrum of rMb(CoCor) in 10 mM ammonium acetate buffer (pH 7.0).

**Figure 6:**
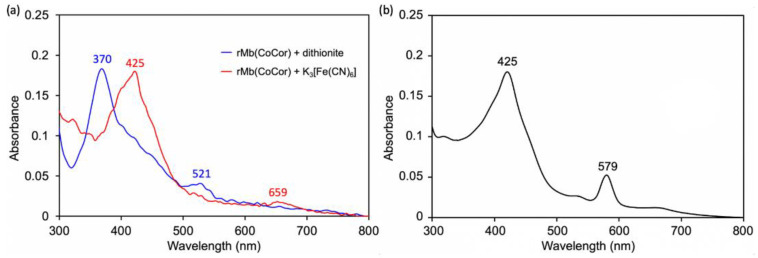
(**a**) UV-vis spectra of rMb(CoCor) containing dithionite (blue line) and potassium ferricyanide (red line). (**b**) UV-vis spectrum of rMb(CoCor) in the presence of 8% pyridine. Conditions: [rMb(CoCor)] = 5 µM in 100 mM potassium phosphate buffer (pH 7.0), 25 °C.

A solution of apoMb prepared according to the previously published procedures [10] was titrated with a pyridine solution of CoCor. Here, pyridine was used to increase the solubility of CoCor in a buffer solution. UV-vis spectral changes and a titration curve monitored by absorbance at 579 nm are shown in Figure 4. The clear conversion of titration curve at one equivalent indicates tight 1:1 complexation of CoCor and apoMb. The solution was purified using a HiTrap^TM^ Desalting column to provide the reconstituted Mb with CoCor, rMb(CoCor). The peak top at 373 nm is similar to the Co octaethylcorrole complex with mono-pyridine axial ligation [17]. This observation indicates that CoCor is coordinated by His93 in the myoglobin matrix. Figure 5 shows the ESI mass spectrum of rMb(CoCor) with corresponding multiply ionized peaks. Peaks attributed to apoMb are also observed but could be generated under the ionization conditions. UV-vis spectra in the titration (Figure 4) are different from that of the purified one (Figure 6a). This is caused by the presence of pyridine because the spectrum observed in the titration is consistent with the purified rMb(CoCor) obtained upon the addition of pyridine (Figure 6b). The spectral changes indicate that pyridine is coordinated to the Co center as a sixth ligand in rMb(CoCor). Thus, pyridine is not coordinated in purified rMb(CoCor) and CoCor in the protein matrix may be in a pentacoordinate state or hexacoordinate state with a water molecule ligand.

**Figure 7:**
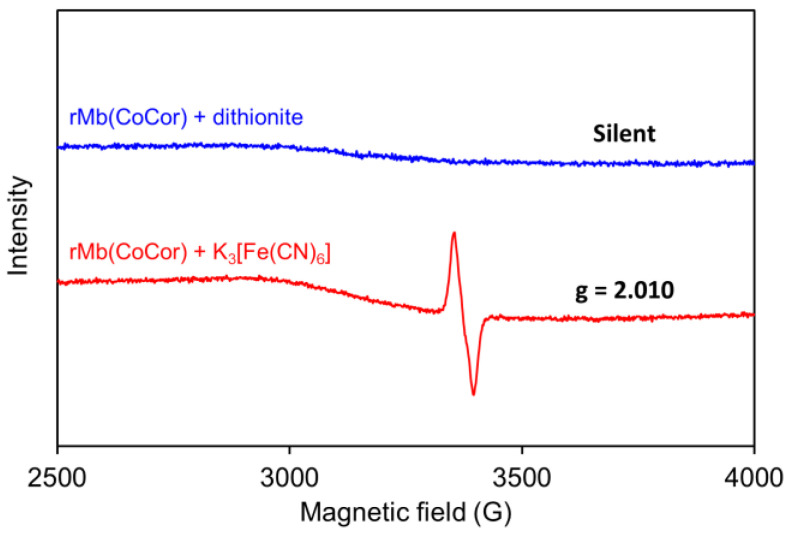
ESR spectra of rMb(CoCor) reduced by dithionite (upper) and rMb(CoCor) oxidized by potassium ferricyanide (bottom). Conditions: [rMb(CoCor)] = 350 μM, [dithionite] = [potassium ferricyanide] = 5 mM in 100 mM potassium phosphate buffer (pH 7.0), MW Power: 2.0 mW, MW Frequency: 9.44 GHz, Mod. Amplitude: 1.0 G, T = 100 K.

To clarify the oxidation state of rMb(CoCor), ESR spectra were evaluated. Dithionite (5 mM) or potassium ferricyanide (5 mM) were added to rMb(CoCor) (350 µM) under a nitrogen atmosphere and equilibrated for 5 min. ESR spectra of the frozen solutions were measured at 100 K (Figure 7). No peak was observed for the reduced state with dithionite, suggesting the presence of a Co^III^ corrole species bound into apoMb, rMb(Co^III^Cor). Dissimilarly, a singlet peak was observed at *g* = 2.010 in the sample oxidized with potassium ferricyanide. The hyperfine structure derived from the Co^IV^ species was not observed, and the singlet peak is characteristic of an organic radical species, indicating that formation of a Co^III^ corrole cation radical species in the Mb matrix (rMb(Co^III^Cor^+^∙)) occurred [18].

**Figure 8:**
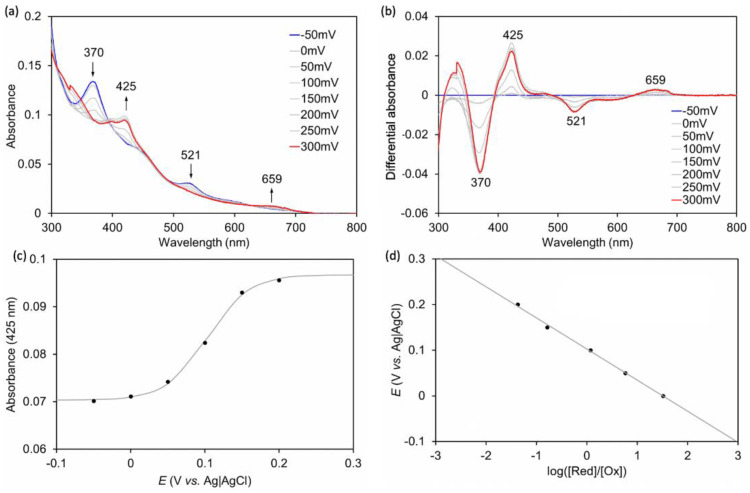
(**a**) UV-vis spectral changes of rMb(CoCor) during applied voltage changing from −50 mV to 300 mV (vs. Ag|AgCl). (**b**) Differential spectra of (**a**). (**c**) Absorption changes at 425 nm in (**a**) against the potentials. (**d**) Nernst plots obtained by spectroelectrochemical measurement of rMb(CoCor). [rMb(CoCor)] = 150 μM, [hydroquinone] = 1.5 mM in 100 mM potassium phosphate buffer (pH 7.0), 25 °C.

Spectroelectrochemical measurements were performed to characterize the redox potential of rMb(CoCor) in 100 mM potassium phosphate buffer solution (pH 7.0). The applied voltage was varied in 50 mV steps from −0.05 V vs. Ag|AgCl to 0.3 V vs. Ag|AgCl at 25 °C under an argon atmosphere, and UV-vis spectra were measured at each step (Figure 8). As a result, it was observed that when the voltage increased positively, the absorption at 370 nm disappeared and the absorption at 425 nm increased. These changes are consistent with the results obtained upon addition of dithionite or potassium ferricyanide, suggesting a change in redox state from rMb(Co^III^Cor) to rMb(Co^III^Cor^+^∙). The absorption changes occurring at 425 nm were analyzed by the Nernst equation to produce a Nernst plot (Figure 8). The plots indicate that the redox potential of rMb(Co^III^Cor)/rMb(Co^III^Cor^+^∙) is 0.10 V vs. Ag|AgCl. The redox potentials of Co^III^Cor/Co^III^Cor^+^∙ in DMF and DMF with 5% pyridine solutions of CoCor are 0.33 and 0.18 V vs. Ag|AgCl, respectively, as stated previously. Although the solvent is different, the redox potential of rMb(CoCor) is negatively shifted compared to redox potentials of the complexes alone. This indicates that the protein matrix stabilizes the oxidized state of CoCor.

### 2.3. Reaction of rMb(CoCor) with Hydrogen Peroxide in the Presence of Phenol Derivatives

**Figure 9:**
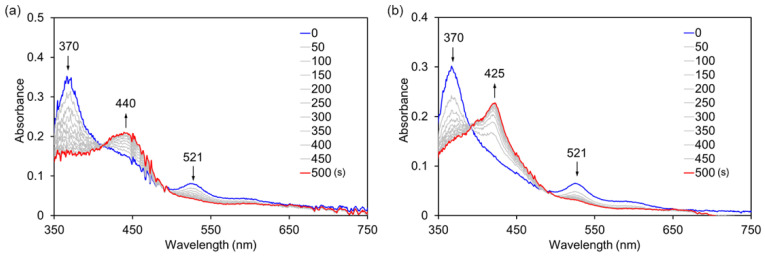
UV-vis spectral changes of rMb(Co^III^Cor) upon addition of H_2_O_2_ without guaiacol (**a**) and with guaiacol (**b**). [rMb(Co^III^Cor)] = 8 μM, [H_2_O_2_] = 10 mM, [guaiacol] = 10 mM in 100 mM potassium phosphate buffer (pH 7.0), 25 °C.

**Scheme 2:**
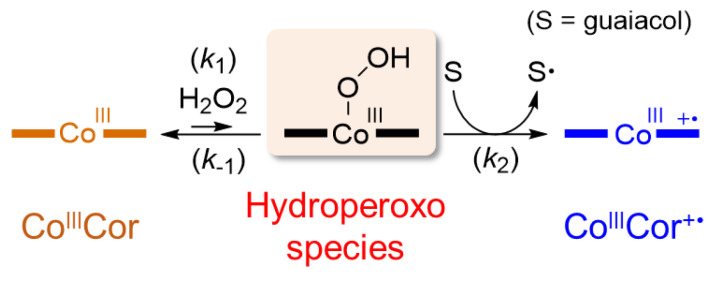
Proposed reaction mechanism of the oxidation reaction with rMb(Co^III^Cor).

In our previous work, it was determined that myoglobin reconstituted with FeCor has much higher catalytic activity than nMb for oxidation of guaiacol (2-methoxyphenol) using hydrogen peroxide [10]. Thus, oxidation activity of rMb(CoCor) for the one-electron oxidation of guaiacol was evaluated. Although the reaction of rMb(Co^III^Cor) with 1 mM guaiacol in the presence of 40 mM hydrogen peroxide provided a slight increase in absorption at 470 nm which is a characteristic absorption for oxidized product of guaiacol, the amount is negligible relative to nMb and rMb(FeCor). To check the reactivity of rMb(Co^III^Cor) with hydrogen peroxide, the transient UV-vis spectral changes of an 8 µM rMb(Co^III^Cor) solution upon addition of 10 mM hydrogen peroxide in the absence and presence of guaiacol were monitored using a stoppered-flow apparatus (Figure 9). Different spectral changes were observed: absorption bands at 370 nm and 521 nm decrease and the absorption band at 440 nm increases in the absence of guaiacol, whereas absorption bands at 370 nm and 521 nm decrease and the absorption band at 425 nm increases in the presence of 10 mM guaiacol. The spectrum obtained in the presence of 10 mM guaiacol is similar to the spectrum of rMb(Co^III^Cor^+^∙), indicating that rMb(Co^III^Cor) reacts with hydrogen peroxide in the presence of substrate and changes to rMb(Co^III^Cor^+^∙). This result suggests that the transiently formed Co^III^ hydroperoxo species in the reaction of rMb(Co^III^Cor) with hydrogen peroxide is an active intermediate in guaiacol oxidation (Scheme 2). On the other hand, the species obtained in the absence of guaiacol is an unknown species, which does not react with guaiacol and dithionite. The ESI mass spectrum of the cofactor has a peak at *m*/*z* = 653.2, which is consistent with *m*/*z* of the oxygen adduct of CoCor. Because it has been reported that the reaction of meso-unsubstituted nickel corrole with oxygen produces a meso-oxidized species [19], the irreversibly oxygenated CoCor at the meso position of the corrole framework appears to be generated by the reaction of rMb(Co^III^Cor) with 10 mM hydrogen peroxide in the absence of guaiacol.

**Figure 10:**
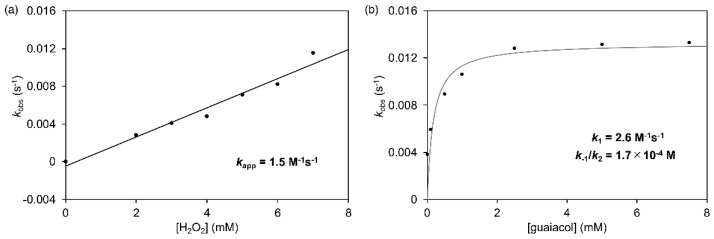
Plots of *k*_obs_ versus concentration of hydrogen peroxide (**a**) and guaiacol (**b**).

To confirm the hypothesis described in Scheme 2, we consider the reaction rate equation (Equation (1)). In this equation, a steady state of Co^III^ hydroperoxo species is assumed in a large excess of hydrogen peroxide and guaiacol relative to rMb(CoCor).

(1) $$k_{obs}=k_{app}\left[H_{2}O_{2}\right]=\frac{k_{1}\left[H_{2}O_{2}\right]}{\frac{k_{-1}}{k_{2}\left[\mathrm{guaiacol}\right]}+1}$$

where *k*_obs_ is an observed pseudo-first-order rate constant to form rMb(Co^III^Cor^+^∙), *k*_app_ is an apparent rate constant in any guaiacol concentration, *k*_1_ is a rate constant to convert rMb(Co^III^Cor) to the hydroperoxo complex, *k_–_*_1_ is a rate constant to convert the hydroperoxo complex to rMb(Co^III^Cor), and *k*_2_ is a rate constant to form rMb(Co^III^Cor^+^∙) in the reaction of the hydroperoxo complex with guaiacol. According to this equation, the reactions of rMb(CoCor) with hydrogen peroxide in the presence of guaiacol were performed using various concentration of hydrogen peroxide and guaiacol. Each reaction process was analyzed by monitoring the transient absorption changes occurring at 425 nm under pseudo-first-order reaction conditions (Figure S1). First, the concentration of hydrogen peroxide was varied from 2 mM to 7 mM in the presence of 10 mM guaiacol. The obtained *k*_obs_ values were plotted for the concentration of hydrogen peroxide as shown in Figure 10a and proportionally increased against hydrogen peroxide concentration. The slope of linear fitting provides *k*_app_ as 1.5 M^−1^s^−1^. Next, the concentration of guaiacol was varied from 0.05 mM to 7.5 mM in the presence of 5 mM hydrogen peroxide. The plots of the *k*_obs_ values against the guaiacol concentration showed saturation (Figure 10b). These plots can be properly fitted to Equation (1) and reveal values of *k*_1_ = 2.6 M^−1^s^−1^ and *k*_–1_/*k*_2_ = 1.7 × 10^−4^ M. Because the hydroperoxo intermediate is not detected in the spectral changes and not detected directly even in the absence of guaiacol, the *k*_1_ value is much smaller than the *k*_–1_ value. Thus, the value of *k*_2_ appears to be greater than 10^4^ M^−1^s^−1^, suggesting that the reaction in the second step is much faster than in the first step in Scheme 2. These results indicate that the formation of Co^III^ hydroperoxo species in the first step is the rate-limiting step and that the Co^III^ hydroperoxo species is an active species for guaiacol oxidation.

**Figure 11:**
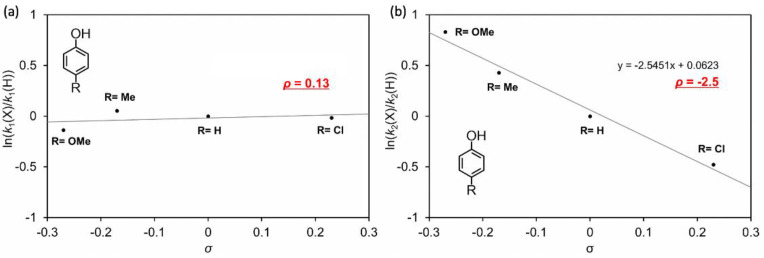
Hammett plots for *k*_1_ (**a**) and *k*_2_ (**b**) in the presence of various phenol derivatives (*p*-methoxy (OMe), *p*-methyl (Me), *p*-chloro (Cl) substituted phenols and unsubstituted phenol).

For further validation of the proposed reaction mechanism, phenol and other phenol derivatives, *p*-methoxyphenol, *p*-methylphenol and *p*-chlorophenol, were employed as reactants. The transient spectral changes observed in the presence of these reactants are similar to the changes observed in the presence of guaiacol, and *k*_1_ and *k*_–1_/*k*_2_ values were determined in the same manner for guaiacol. The values are summarized in Table 1 and plotted against Hammett substituent constants, *σ* (Figure 11), where *k*_–1_ is assumed to be independent of reactant [20]. The slopes provide Hammett reaction constants, *ρ*: *ρ* for *k*_1_ is 0.13 and *ρ* for *k*_2_ is −2.5. The *k*_1_ values are mostly independent of reactant substituents and *k*_2_ values strongly depend on the reactant substituent. These results are consistent with the proposed reaction mechanism. The *ρ* for *k*_2_ is negative, showing acceleration for electron-donating substituents and a rate-limiting step of one electron transfer from phenol derivatives to the hydroperoxo species [21]. These findings support the proposed reaction mechanism for oxidation of phenol derivatives.

**Figure 12:**
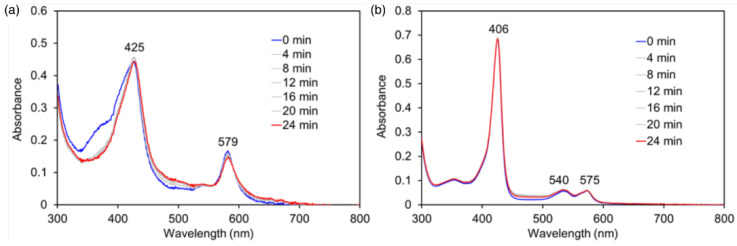
UV-vis spectral changes upon addition of H_2_O_2_ to a solution of CoCor containing 5% *v/v* pyridine (**a**) and rMb(CoPor) (**b**) in the presence of guaiacol. [CoCor] = [rMb(CoPor)] = 8 μM, [H_2_O_2_] = 10 mM, [guaiacol] = 10 mM in 100 mM potassium phosphate buffer (pH 7.0), 25 °C.

The reaction of CoCor with hydrogen peroxide in the presence of guaiacol was performed to investigate the effect of the protein matrix. No significant spectral changes were observed in the presence of 5% pyridine (Figure 12a). This result suggests that CoCor does not promote substrate oxidation reactions. The protein matrix appears to be important for the formation of the hydroperoxo species. A similar experiment was performed using reconstituted myoglobin with a Co porphyrin complex (rMb(CoPor)) (Figure 12b). Further, in this case, no spectral changes were also observed. One of the reasons for the observed higher oxidation activity of rMb(CoCor) compared to rMb(CoPor) is due to the stabilization of the formal Co^IV^ species formed by the corrole ligand which may improve the reactivity of the transiently formed hydroperoxo active species.

Bleomycin, a natural antibiotic of the glycopeptide family, forms an Fe^II^ complex to catalyze oxidative cleavage of DNA via the hydroperoxo complex as an active intermediate [22]. In this case, the formation of the oxo species is energetically unfavorable. Inspired by this system, oxidation reactions with the metal hydroperoxo species as active oxidizing species have been reported. For example, W. Nam and coworkers demonstrated that the Fe^III^ hydroperoxo complex of tetramethyl-1,4,8,11-tetraazacyclodecane serves as an effective active oxidizing species in alkane C–H bond activation [23]. Solomon and coworkers reported that the Co^III^ hydroperoxo complex of 3,6,9-trimethyl-3,6,9-triaza-1(2,6)-pyridinacyclodecaphane promotes electrophilic oxygen atom transfer reactions for thioanisole and triphenylphosphine [24]. In this context, our present work is the first demonstration of a hydroperoxo Co^III^ species promoting an oxidation reaction in a protein matrix.

## 3. Conclusions

A Co corrole complex was synthesized and incorporated into the apo-form of myoglobin to produce reconstituted myoglobin. The reconstituted protein has the characteristic reactivity for hydrogen peroxide in the presence of phenol derivatives; a transiently formed hydroperoxo species oxidizes the phenol derivatives. This mechanism is quite different from the reaction mechanism of native heme-dependent peroxidases such as horseradish peroxidase, which first generates compound I, an Fe^IV^-oxo porphyrin π-cation radical species, as an active intermediate in its reaction with hydrogen peroxide. This difference of the reaction mechanism is a result of an “oxo-wall”: the inability of the complex to form the Co-oxo species [25]. In the recently developed field of artificial metalloenzymes, various reactions are utilized for further progress [26,27,28,29] and unique applications [30,31,32]. Thus, the unique findings provided by our investigation of catalytic oxidation of the phenol derivatives with hydrogen peroxide are expected to contribute to further design of artificial metalloenzymes.

## 4. Materials and Methods

### 4.1. Instruments

UV-vis spectral measurements were carried out with a UV-3150 or UV-2550 double-beam spectrophotometer (Shimadzu, Kyoto, Japan) or a BioSpec-nano spectrometer (Shimadzu). ESI-TOF MS analyses were performed with a micrOTOF-II mass spectrometer (Bruker, Billerica, MA, USA). ^1^H NMR spectra were collected on an Avance III HD (400 MHz) spectrometer (Bruker). The ^1^H NMR chemical shift values are reported in ppm relative to a residual solvent peak. EPR spectra were measured using an EMX Micro spectrophotometer (Bruker). pH measurements were carried out with an F-72 pH meter (Horiba, Kyoto, Japan). ICP-OES was performed on an ICPS-7510 emission spectrometer (Shimadzu). The kinetic measurements were conducted with a rapid scan stopped-flow system (Unisoku, Hirakata, Japan) constructed with a Xe or halogen light source.

### 4.2. Materials

All chemicals were purchased from Wako, TCI, Nacalai, and Sigma-Aldrich and were used as received unless otherwise noted. [*a,c*]-Biladiene salt **1** was synthesized as described in the previous literature [10]. Removal of heme from nMb and preparation of apoMb were performed according to reported procedures [10]. CoCor and rMb(CoCor) were prepared as described below.

### 4.3. Synthesis of CoCor

#### 4.3.1. Synthesis of Corrole **2**

[*a,c*]-Biladiene salt **1** (250 mg, 0.323 mmol) and sodium acetate (500 mg, 6.10 mmol) were dissolved in methanol (50 mL) and refluxed for 3 h. The solvent was removed under a reduced pressure and the resulting solid was redissolved in chloroform. After washing with distilled water and saturated brine, the solid was dried with sodium sulfate and the solvent was removed under a reduced pressure. The product was purified by silica gel column chromatography (chloroform:ethyl acetate = 9:1), and the purple fraction was collected. The solvent was removed by evaporation and the resulting solid was washed with hexane to afford corrole **2** as a purple solid (40 mg, 60.6 µmol, 19%).

ESI-TOF MS (positive mode, methanol) *m*/*z* calcd for C_37_H_46_N_4_O_4_ [M]^+^ 611.35, found 611.33

^1^H NMR (400 MHz, CDCl_3_)

*δ* (ppm) = 9.38 (s, 2H), 9.18 (s, 1H), 4.22 (t, 4H, *J* = 7.9 Hz), 3.98–3.88 (q, 4H, *J* = 7.8 Hz), 3.69 (s, 6H), 3.45 (s, 6H), 3.19 (t, 4H, *J* = 7.9 Hz), 1.77–1.72 (t, 12H, *J* = 7.8 Hz)

#### 4.3.2. Synthesis of Co Corrole Complex **3**

Corrole **2** (20 mg, 32.8 µmol), triphenylphosphine (PPh_3_: 40 mg, 152 µmol), and Co^II^ acetate tetrahydrate (40 mg, 161 µmol) were dissolved in methanol/chloroform = 1:1 mixed solvent (40 mL) and refluxed for 2 h. The product was extracted with chloroform, washed with saturated brine, dried over sodium sulfate, and the solvent was removed under a reduced pressure. The residue was purified via silica gel column chromatography (chloroform:ethyl acetate = 10:1), and the colored fraction was collected. The solvent was removed under a reduced pressure to yield Co corrole complex **3** as a red solid (20.0 mg, 21.6 µmol, 66%).

ESI-TOF MS (positive mode, methanol) *m*/*z* calcd for C_55_H_58_N_4_O_4_PCo [M]^+^ 928.3522, found 928.3537

^1^H NMR (400 MHz, CDCl_3_ containing 1% D_2_O)

*δ* (ppm) = 9.32 (s, 1H), 9.06 (s, 2H), 7.69–7.44 (m, 15H), 3.90 (t, 4H, *J* = 7.8 Hz), 3.69 (s, 6H), 3.64 (m, 8H), 3.14 (s, 6H), 2.96 (t, 4H, *J* = 7.8 Hz), 1.61–1.59 (tt, 12H, *J* = 7.6 Hz)

#### 4.3.3. Synthesis of CoCor

Co corrole complex **3** (15.0 mg, 16.2 µmol) was dissolved in a methanol/THF = 1:1 mixture (50 mL) and potassium hydroxide solution (0.5 M, 50 mL) was added dropwise over 30 min at room temperature under a nitrogen atmosphere. After stirring at room temperature for 12 h, the pH was adjusted to ca. 6 upon addition of 10% citric acid solution. The mixture was extracted with chloroform, washed with saturated brine, dried with sodium sulfate, and the solvent was removed under a reduced pressure. The product was purified via gel filtration chromatography (Sephadex LH-20, methanol) to afford CoCor as a red solid (12.8 mg, 14.2 µmol, 88%).

ESI-TOF MS (positive mode) *m*/*z* calcd for C_53_H_54_N_4_O_4_PCo [M]+ 900.3209, found 900.3216

### 4.4. Reconstitution of Mb with CoCor

A pyridine solution of CoCor (500 µM) was slowly added dropwise to the apoMb solution (20 µM, 10 mL). The solution was allowed to equilibrate at room temperature for 5 min, and the 1:1 complexation of apoMb and CoCor was confirmed by noting the absorption changes at 579 nm in the UV-vis spectrum. After equilibration at 4 °C for 2 h, the solution was concentrated and the solution was purified on a HiTrap^TM^ Desalting column (GE healthcare, 100 mM potassium phosphate buffer, pH 7.0) to afford rMb(CoCor) (60 µM, 1.5 mL, 45%). The extinction coefficient was determined through ICP measurement: *ε* at 390 nm = 36,000 M^−1^cm^−1^.

### 4.5. Cyclic Voltammetry

Cyclic voltammetry measurements were performed in a DMF solution containing Co corrole complex **3** (0.5 mM) and Bu_4_NPF_6_ salt (100 mM) under a nitrogen atmosphere (scan rate: 100 mV/s, glassy carbon working electrode, Ag|AgCl standard electrode, Pt counter electrode). The results obtained show the potentials calibrated via ferrocene cyclic voltammetry measurements. The potential was swept over the range from –2.19 V to 0.59 V vs Ag|AgCl.

### 4.6. Kinetic Analysis

According to Scheme 2, the Equation (2)–(4) were developed.

(2) $$\frac{d\left[\mathrm{rMb}\left({\mathrm{Co}}^{\mathrm{III}}\mathrm{Cor}\right)\right]}{\mathrm{dt}}=-k_{1}\left[H_{2}O_{2}\right]\left[\mathrm{rMb}\left({\mathrm{Co}}^{\mathrm{III}}\mathrm{Cor}\right)\right]+k_{-1}\left[H_{2}O_{2}\right]\left[\mathrm{rMb}\left(\mathrm{HOO}-{\mathrm{Co}}^{\mathrm{III}}\mathrm{Cor}\right)\right]$$

(3) $$\frac{d\left[\mathrm{rMb}\left(\mathrm{HOO}-{\mathrm{Co}}^{\mathrm{III}}\mathrm{Cor}\right)\right]}{\mathrm{dt}}=k_{1}\left[H_{2}O_{2}\right]\left[\mathrm{rMb}\left({\mathrm{Co}}^{\mathrm{III}}\mathrm{Cor}\right)\right]-\left(k_{-1}+k_{2}\left[\mathrm{guaiacol}\right]\right)\left[\mathrm{rMb}\left(\mathrm{HOO}-{\mathrm{Co}}^{\mathrm{III}}\mathrm{Cor}\right)\right]$$

(4) $$\left[\mathrm{rMb}\left({\mathrm{Co}}^{\mathrm{III}}\mathrm{Cor}\right)\right]+\left[\mathrm{rMb}\left({\mathrm{Co}}^{\mathrm{III}}{\mathrm{Cor}}^{+\cdot }\right)\right]={\left[\mathrm{rMb}\left({\mathrm{Co}}^{\mathrm{III}}\mathrm{Cor}\right)\right]}_{0}$$

where rMb(HOO-Co^III^Cor) is a hydroperoxo complex.

Here, we assumed that rMb(HOO–Co^III^Cor) is in a steady state

(5) $$\frac{d\left[\mathrm{rMb}\left(\mathrm{HOO}-{\mathrm{Co}}^{\mathrm{III}}\mathrm{Cor}\right)\right]}{\mathrm{dt}}=0$$

and from Equation (3)

(6) $$\left[\mathrm{rMb}\left(\mathrm{HOO}-{\mathrm{Co}}^{\mathrm{III}}\mathrm{Cor}\right)\right]=\frac{k_{1}\left[H_{2}O_{2}\right]\left[\mathrm{rMb}\left({\mathrm{Co}}^{\mathrm{III}}\mathrm{Cor}\right)\right]}{k_{-1}+k_{2}\left[\mathrm{guaiacol}\right]}$$

Substituting this into Equation (2), we obtain

(7) $$\frac{d\left[\mathrm{rMb}\left({\mathrm{Co}}^{\mathrm{III}}\mathrm{Cor}\right)\right]}{dt}=\frac{k_{1}\left[H_{2}O_{2}\right]\left[\mathrm{rMb}\left({\mathrm{Co}}^{\mathrm{III}}\mathrm{Cor}\right)\right]}{\frac{k_{-1}}{k_{2}\left[\mathrm{guaiacol}\right]}+1}$$

Assuming that [H_2_O_2_], [guaiacol] is in large excess with respect to [rMb(CoCor)], the following is written in terms of the pseudo-first-order rate constant *k*_obs_:

(8) $$\frac{d\left[\mathrm{rMb}\left({\mathrm{Co}}^{\mathrm{III}}\mathrm{Cor}\right)\right]}{dt}=-k_{obs}\left[\mathrm{rMb}\left({\mathrm{Co}}^{\mathrm{III}}\mathrm{Cor}\right)\right]$$

From Equations (7) and (8), Equation (1) is provided.

## Figures and Tables

**Table 1:** Kinetic parameters for the reaction of rMb(Co^III^Cor) with hydrogen peroxide in the presence of phenol and its derivatives ^1^.

Reactant	*k*_1_ (M^−1^s^−1^)	*k*_–1_/*k*_2_ (M)
Phenol	3.4	6.5 × 10^−4^
*p*-methoxyphenol	3.0	2.8 × 10^−4^
*p*-methylphenol	3.6	4.2 × 10^−4^
*p*-chlorophenol	3.3	1.0 × 10^−3^

^1^ [rMb(Co^III^Cor)] = 8 µM, [H_2_O_2_] = 5 mM in 100 mM potassium phosphate buffer (pH 7.0) at 25 °C.

## Supplementary Materials

The following supporting information can be downloaded at: https://www.mdpi.com/article/10.3390/ijms23094829/s1.
