# Supplementary material for: Reactivity of Myoglobin Reconstituted with Cobalt Corrole toward Hydrogen Peroxide
Source: Int J Mol Sci. 2022 Apr 27;23(9):4829. doi: 10.3390/ijms23094829 (PMC9104730; doi:10.3390/ijms23094829)
Supplement: Supplementary file 1 [file ijms-23-04829-s001.zip › ijms-1695263-supplementary.pdf]

Reactivity of myoglobin reconstituted with cobalt corrole toward hydrogen peroxide

Koji Oohora,*Takahiro Tomoda, and Takashi Hayashi*

ELECTRONIC SUPPLEMENTARY INFORMATION

Table of contents:

1. Figure S1. Transient absorption changes of rMb(Co^{III}Cor) upon addition of various concentration of H_2O_2 in the presence of 10 mM guaiacol.
2. Figure S2. Transient absorption changes of rMb(Co^{III}Cor) upon addition of 5 mM H_2O_2 in the presence of various concentration of guaiacol.

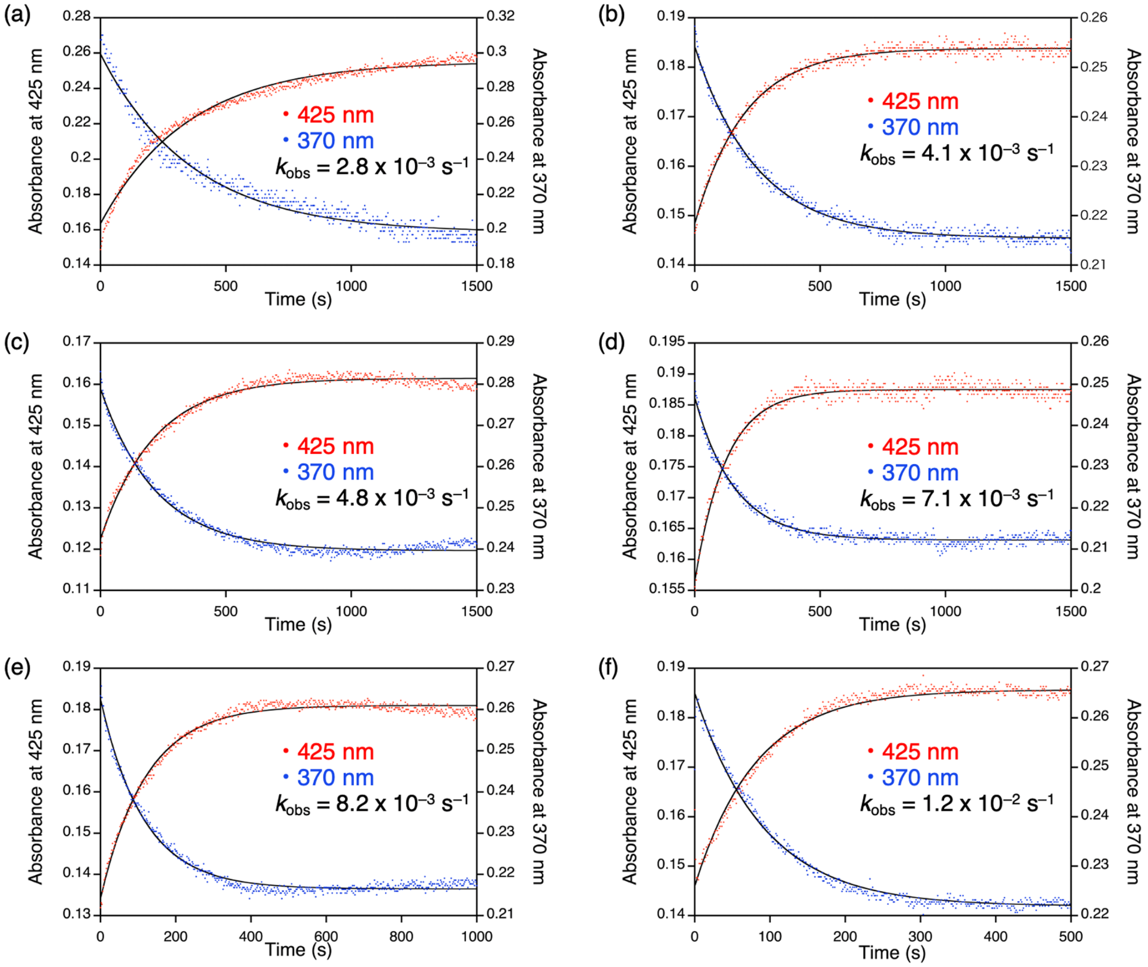

Figure S1: Transient absorption changes of rMb(Co^{III}Cor) upon addition of various concentration of H_2O_2 in the presence of 10 mM guaiacol. The solid lines show fitting curves by an equation of $Abs(t) = Abs_\infty + Abs_0 \times \exp(-k_{obs} \times t)$, where Abs_∞ and Abs_0 are absorbances at $t = \infty$ and $t = 0$, respectively. Conditions: [rMb(Co^{III}Cor)] = 8 μM; [H_2O_2] = 2 mM (a), 3 mM (b), 4 mM (c), 5 mM (d), 6 mM (e), or 7mM (f); [guaiacol] = 10 mM in 100 mM potassium phosphate buffer (pH 7.0) at 25 °C.

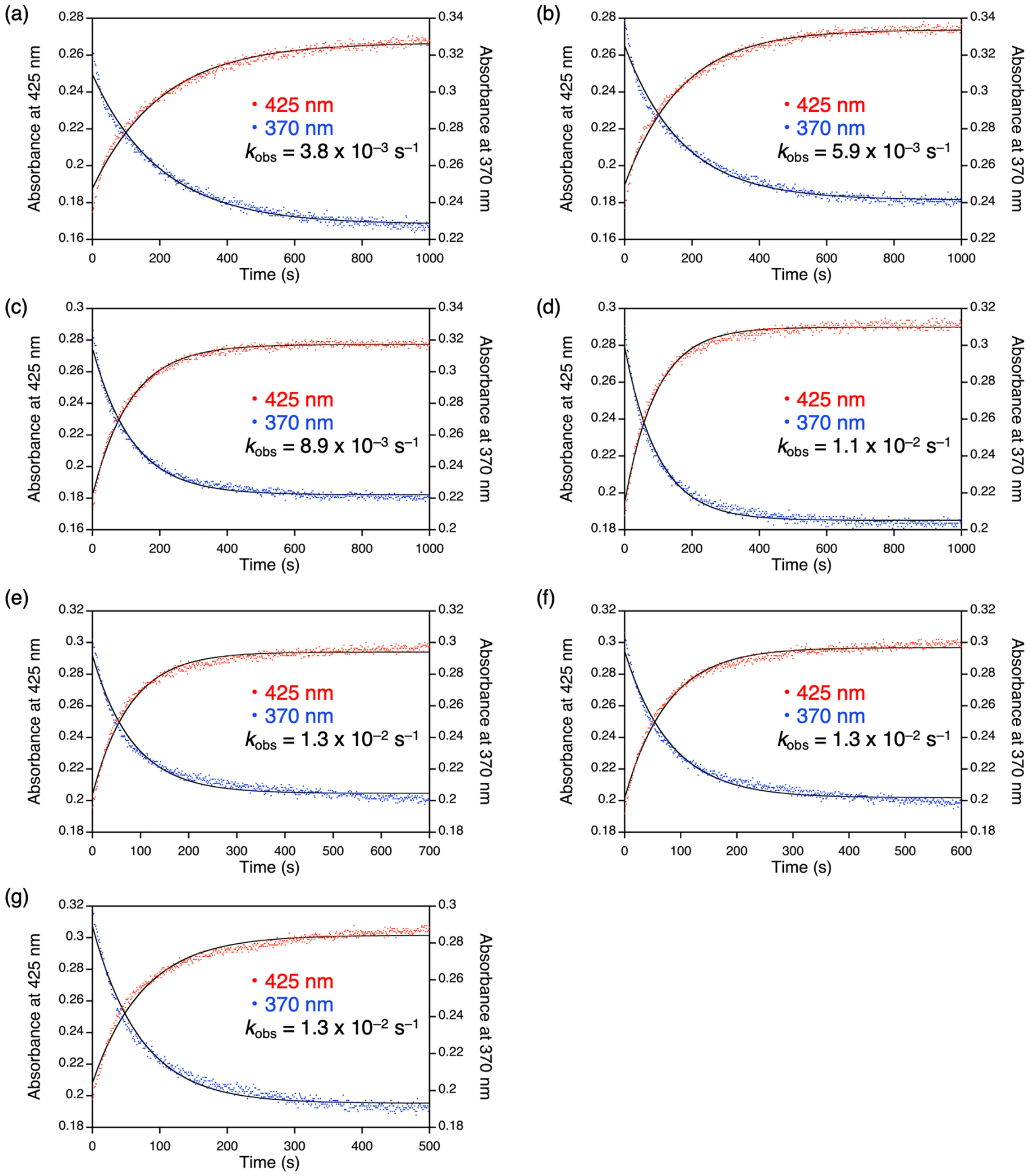

Figure S2. Transient absorption changes of rMb(Co^{III}Cor) upon addition of 5 mM H_2O_2 in the presence of various concentration of guaiacol. The solid lines show fitting curves by an equation of $Abs(t) = Abs_{\infty} + Abs_0 \times \exp(-k_{obs} \times t)$, where Abs_{∞} and Abs_0 are absorbances at $t = \infty$ and $t = 0$, respectively. Conditions: [rMb(Co^{III}Cor)] = 8 μM; [H_2O_2] = 5 mM; [guaiacol] = 0.05 mM (a), 0.1 mM (b), 0.5 mM (c), 1.0 mM (d) 2.5 mM (e), 5.0 mM (f), or 7.5 mM (g) in 100 mM potassium phosphate buffer (pH 7.0) at 25 °C.
